# Biochemical Characterization of a Haloalkane Dehalogenase DadB from *Alcanivorax dieselolei* B-5

**DOI:** 10.1371/journal.pone.0089144

**Published:** 2014-02-28

**Authors:** Anzhang Li, Zongze Shao

**Affiliations:** 1 School of Life Sciences, Xiamen University, Xiamen, China; 2 Key Laboratory of Marine Biogenetic Resources—State Key Laboratory Breeding Base, Third Institute of Oceanography, State Oceanic Administration, Collaborative Innovation Center of Deep Sea Biology; Key Laboratory of Marine Biogenetic Resources of Fujian Province, Xiamen, China; Universidade Nova de Lisboa, Portugal

## Abstract

Recently, we found that *Alcanivorax* bacteria from various marine environments were capable of degrading halogenated alkanes. Genome sequencing of *A. dieselolei* B-5 revealed two putative haloalkane dehalogenase (HLD) genes, which were supposed to be involved in degradation of halogenated compounds. In this report, we confirm for the first time that the *Alcanivorax* bacterium encodes a truly functional HLD named DadB. An activity assay with 46 halogenated substrates indicated that DadB possesses broad substrate range and has the highest overall activity among the identified HLDs. DadB prefers brominated substrates; chlorinated alkenes; and the C_2_-C_3_ substrates, including the persistent pollutants of 1,2-dichloroethane, 1,2-dichloropropane and 1,2,3-trichloropropane. As DadB displays no detectable activity toward long-chain haloalkanes such as 1-chlorohexadecane and 1-chlorooctadecane, the degradation of them in *A. dieselolei* B-5 might be attributed to other enzymes. Kinetic constants were determined with 6 substrates. DadB has highest affinity and largest *k*_cat_/*K*_m_ value toward 1,3-dibromopropane (*K*_m_ = 0.82 mM, *k*_cat_/*K*_m_ = 16.43 mM^−1^·s^−1^). DadB aggregates fast in the buffers with pH≤7.0, while keeps stable in monomer form when pH≥7.5. According to homology modeling, DadB has an open active cavity with a large access tunnel, which is supposed important for larger molecules as opposed to C_2_-C_3_ substrates. Combined with the results for other HLDs, we deduce that residue I247 plays an important role in substrate selection. These results suggest that DadB and its host, *A. dieselolei* B-5, are of potential use for biocatalysis and bioremediation applications.

## Introduction

Bacteria of the genus *Alcanivorax* are widespread in marine environments as important alkane degrader [1]. Recently, we found that *Alcanivorax* was the dominant group capable of 1-chlorohexadecane degradation in the surface water of the Arctic [2]. *Alcanivorax dieselolei* strain B-5 was first isolated from the surface water of the Bohai Sea [3]. It has multiple alkane hydroxylase systems to ensure that it can utilize a wide range of alkanes with different chain lengths (C_5_-C_36_) [4]. Strain B-5 was also able to utilize various haloalkanes with short to long chains as the sole carbon source [5]. However, the mechanism through which *Alcanivorax* degrades haloalkane substrates remains undetermined.

Haloalkane dehalogenases (HLDs) are key enzymes for degrading halogenated aliphatic pollutants [6]–[8]. They belong to the α/β-hydrolase superfamily and cleave the carbon-halogen bond through hydrolysis [9], [10]. However, only a few HLDs have been proved to ensure their soil bacteria hosts to utilize such compounds [11]–[15]. Recently, during our genome sequencing analysis of *A. dieselolei* B-5, two putative HLD genes were identified [16], which may be responsible for degrading haloalkane substrates.

Based on phylogenetic analyses, HLDs can be divided into three subfamilies, HLD-I, HLD-II and HLD-III [17]. These subfamilies employ different forms of the catalytic pentad, which is essential for catalytic activity [10]. To date, most characterized HLDs belong to the HLD-II subfamily, which was segregated first from the ancestors of HLD-I and HLD-III [17]. In addition, although a few putative HLD genes exist in eukaryotes, the HLDs that have been characterized experimentally are all derived from bacteria.

Different HLDs showed different substrate selectivity and activity characteristics. Koudelakova et al. selected a set of 30 typical substrates to determine the substrate specificity profiles of HLDs and accordingly classified HLDs into four specificity subfamilies [18]. However, the specificity subfamilies do not correspond with the phylogenetic subfamilies. The authors suggest that the architecture of the active site and the access tunnel, rather than sequence homology, is more important for substrate specificity of HLDs.

HLDs have a conserved structure that is composed of a core domain and a cap domain, between which the active site is located [10]. HLDs have two adjacent access tunnels (the main tunnel and the slot tunnel) to achieve the exchange of substrates, products and ligands between the hydrophobic active site and the surrounding solvent [19]–[22]. The access tunnels play an important role in substrate specificity, catalytic activity and enantioselectivity [19], [23], [24]. For example, narrowing the main tunnel results in a preference for small substrates [25].

Although the biological function of many characterized HLDs remains unknown [26]–[30], they have displayed potential for many applications, such as bioremediation [31], [32], decontamination of warfare agents [33], biosensing [34], biocatalysis [24], [35], cell imaging [36], and protein analysis [37], [38].

In this work, we biochemically characterized a new HLD, named DadB, in an important marine oil-degrading bacterium *A. dieselolei* B-5 and evaluated its optimum reaction conditions, substrate specificity, kinetic constants and stability. Furthermore, the relationship of its structure and function was also documented based on homology modeling.

## Materials and Methods

### Bacterium and chemicals

*A. dieselolei* B-5^T^ was isolated and identified as a novel species by our group [3], and it has been deposited in the Marine Culture Collection of China (MCCC, Xiamen, China) (MCCC1A00001). The 46 halogenated substrates used in the activity determination were purchased from Sigma-Aldrich (MO, U.S.A.), TCI (Japan), Alfa Aesar (MA, U.S.A.), SCRC (Sinopharm Chemical Reagent Co., Ltd, China), and Dr. Ehrenstorfer GmbH (Germany) and are listed in Table S1 of File S1.

### Gene cloning and vector construction

The PCR primers for *dadA* and *dadB* (Table S2 in File S1) were designed according to their sequences within the B-5 genome (YP_006819020) to satisfy an optimized ligation-independent cloning (LIC) method [39]. The expression vectors pET22b-*dadB*, pET22b-*dadA*, pET28a-*dadA*, pET32a-*dadA* and pGEX-4T-1-*dadA* were constructed with amplified *dadB* or *dadA* genes and linearized vectors based on the LIC strategy. DNA sequencing confirmed that no mutation occurred in the inserted open reading frames.

### Expression and purification

pET22b-*dadA*, pET28a-*dadA*, pET32a-*dadA* and pGEX-4T-1-*dadA* were transformed into *E. coli* BL21(DE3), *E. coli* Rosetta(DE3), and *E. coli* Rosetta-gemi(DE3) competent cells and expressed in lysogeny broth (LB) medium or auto-inducing medium (AIM) (24 g of yeast extract, 12 g of peptone, 5 g of NaCl, 5 g of sodium succinate, 20 mL of glycerol, 5 g of lactose, 5.88 g of trisodium citrate dihydrate, 6.8 g of KH_2_PO_4_, 17.91 g of Na_2_HPO_4_•12 H_2_O, add water to 1 L, pH 7.4; sterilized by autoclaving for 15 min; glucose and MgSO_4_ stock solutions were added to final concentrations of 0.5 g/L and 1 mM, respectively, before inoculation) under different inducing temperatures.

pET22b-*dadB* was transformed into *E. coli* BL21(DE3) competent cells. After culturing in LB, the seed culture was inoculated into AIM and cultured at 37°C, 250 rpm for 6 h. When an OD_600_ of 3 was reached, the culture was cooled to 25°C and cultured for additional 14 h.

For DadA purification, ultrasonic disruption buffers with different pH and additives were tried. Metal-affinity chromatography under native and denaturing conditions, GST-affinity purification, and ion-exchange chromatography were optimized.

To purify DadB with His tag, binding buffer (50 mM Tris, 200 mM Na_2_SO_4_, 20 mM imidazole, 10% glycerol, pH 8.5) was added at a ratio of 10 mL/g wet weight biomass to thoroughly suspend the harvested bacteria. After ultrasonic disruption and centrifugation, Ni Sepharose 6 Fast Flow (GE Healthcare, NJ), which was pre-equilibrated with binding buffer and precooled to 4°C, was added to the supernatant, and the samples were incubated at 4°C for 30 min. Then, the mixture was transferred into a simple gravity flow column. After washing successively with binding buffer and washing buffer (50 mM Tris, 200 mM Na_2_SO_4_, 50 mM imidazole, pH 8.5), the target protein was eluted with elution buffer (50 mM Tris, 200 mM Na_2_SO_4_, 160 mM imidazole, pH 8.5) and dialyzed against 50 mM KH_2_PO_4_, pH 7.5 for 3 times, for 6–8 h each time. After concentrating, the purified enzyme was cryopreserved in 50 mM KH_2_PO_4_, 1 mM β-mercaptoethanol, and 10% glycerol (pH 7.5) at −74°C. The above steps were completed at 4°C or on ice. The purification process was assessed by SDS-PAGE, and band analysis was conducted by Quantity One software (Bio-Rad, CA).

### Enzymatic activity assay

The activity of DadB toward 46 substrates, including 30 typical substrates selected by Koudelakova et al., was determined with an end-point spectrophotometric assay [18]. The 5 mL reaction system was prepared in headspace vials with 100 mM glycine buffer, pH 8.6. Then, the substrate and DadB were successively added to a final concentration of 10 mM and 0.01 mg/mL, respectively. The vial was sealed quickly and put into oscillatory water bath at 37°C. The reaction solution was sampled from the system using a syringe at different times. Each 500 µL sample was immediately mixed with 50 µL 30% nitric acid to terminate the reaction. Then, 55 µL of mercuric thiocyanate and 110 µL of ferric ammonium sulfate were added successively. Each mixed solution was transferred into 3 wells of a 96-well plate, and the absorbance at 460 nm was detected using a SpectraMax M5 Microplate Reader (Molecular Devices, CA). An abiotic control was run without DadB to test the spontaneous hydrolysis of substrates under the same conditions. The activity determination for each substrate was repeated at least three times.

### The effects of temperature and pH on DadB activity

To obtain the optimal temperature, dehalogenation activities were measured in triplicate with 1,3-dibromopropane over the range 20–60°C, with intervals of 5°C, in 100 mM glycine buffer, pH 8.6. To test the effects of pH, five sets of buffers (100 mM) with overlapped pH ranges were used to measure DadB's activities in triplicate: potassium acetate with pH 4.0–6.0; KH_2_PO_4_ with pH 6.0–8.0; glycine buffer with pH 8.0–10.0; MOPS with pH 6.0–8.0; and imidazole with pH 5.5–9.0.

### Dynamic light scattering analysis

DadB was diluted into different buffers (100 mM potassium acetate buffer with pH 5.0, 5.5, or 6.0; 100 mM KH_2_PO_4_ with pH 6.0, 6.5, 7.0, 7.5, or 8.0; or 100 mM glycine buffer with pH 8.0, 8.5, 9.0, or 10.0), with a final concentration of 0.87 mg/mL. After centrifugation at 4°C and 20,000 *g* for 10 min, the supernatant was placed into the sample chamber of a dynamic light scatter (Malvern Zetasizer Nano ZS, Malvern, UK). After prewarming for 2 min, each sample was measured 10 times, each measurement took 2 min at 37°C.

### Determination of steady-state kinetic constants

The steady-state kinetic constants of DadB toward 6 substrates were measured. The reaction conditions were the same as for activity determination, except 500 µL of methanol was used to terminate the reaction. The concentration of substrates and products were determined with a gas chromatograph (GC-2010, Shimadazu, Japan) equipped with a flame ionization detector and Agilent DB-FFAP column (30 m×0.25 mm×0.25 µm) (J&W Scientific, CA). The inlet and the detector were 240°C and 250°C, respectively, and the split ratio was 50∶1. Helium was used as the carrier gas, and the flow rate was 1 mL/min. The column temperature for each substrate was constant (Table S3 in File S1). Kinetic equation fitting was conducted using GraphPad Prism 5.01 (GraphPad Software, CA).

### Homology modeling

Homology modeling was conducted in the SWISS-MODEL Web Server under “Automated Mode.” The server selected LinB (PDB code 1iz7), which has 62% similarity with DadB, as the template to construct the DadB model.

### Nucleotide sequence accession number

Complete genome information for *A. dieselolei* B-5 (GenBank: CP003466.1) was submitted to the NCBI Genome Database [16]. The accession numbers of *dadA* and *dadB* in NCBI are YP_006820866.1 and YP_006819020.1, respectively.

## Results and Discussion

### *dadA* and *dadB*, two putative HLD genes, in strain B-5

Two annotated α/β-hydrolase genes, *dadA* and *dadB*, from the complete genome sequence of strain B-5 were previously annotated as HLD genes [16]. The open reading frames of *dadA* and *dadB* consist of 885 and 888 nucleotides, encoding two proteins of 294 and 295 amino acids in length, respectively.

A BLAST search for non-redundant (nr) protein sequences within the NCBI database with DadA and DadB resulted in numerous hits, which were mostly annotated “haloalkane dehalogenase” or “α/β-hydrolase” and included characterized HLDs, such as LinB, DhaA and DmbA. The first hit for DadA is a putative HLD (YP_007362382.1) from *Myxococcus stipitatus* DSM 14675, with a sequence identity of 44%, whereas DadB showed the highest sequence identity (71%), with a putative HLD (ZP_05095081.1) from marine γ-proteobacterium HTCC2148. Among the HLDs that have been biochemically characterized (Table S4 in File S1), DadA and DadB showed the highest homology with DhaA (38% identity) from *Rhodococcus rhodochrous* NCIMB 13064 and LinB (62% identity) from *Sphingobium japonicum* UT26, respectively.

Multiple sequence alignment revealed that DadA and DadB had the typical catalytic pentad of the HLD-II subfamily (Figure S1 in File S1). The phylogenetic analysis indicated that DadB likely belonged to the HLD-II subfamily, closely related with LinB and DmbA, while DadA appeared to be relatively independent from the HLD-II subfamily (Figure S2 in File S1).

Because the information suggests that DadA and DadB are putative HLDs, they were deduced to be involved in the degradation of halogenated compounds. Then, both genes were subjected to protein expression. However, DadA was always expressed in the form of inclusion bodies in *E. coli*, and it was hard to obtain soluble enzyme. Thus, only DadB was subjected to further analyses in this report.

### Heterogeneous expression and purification of DadB

**Figure 1:**
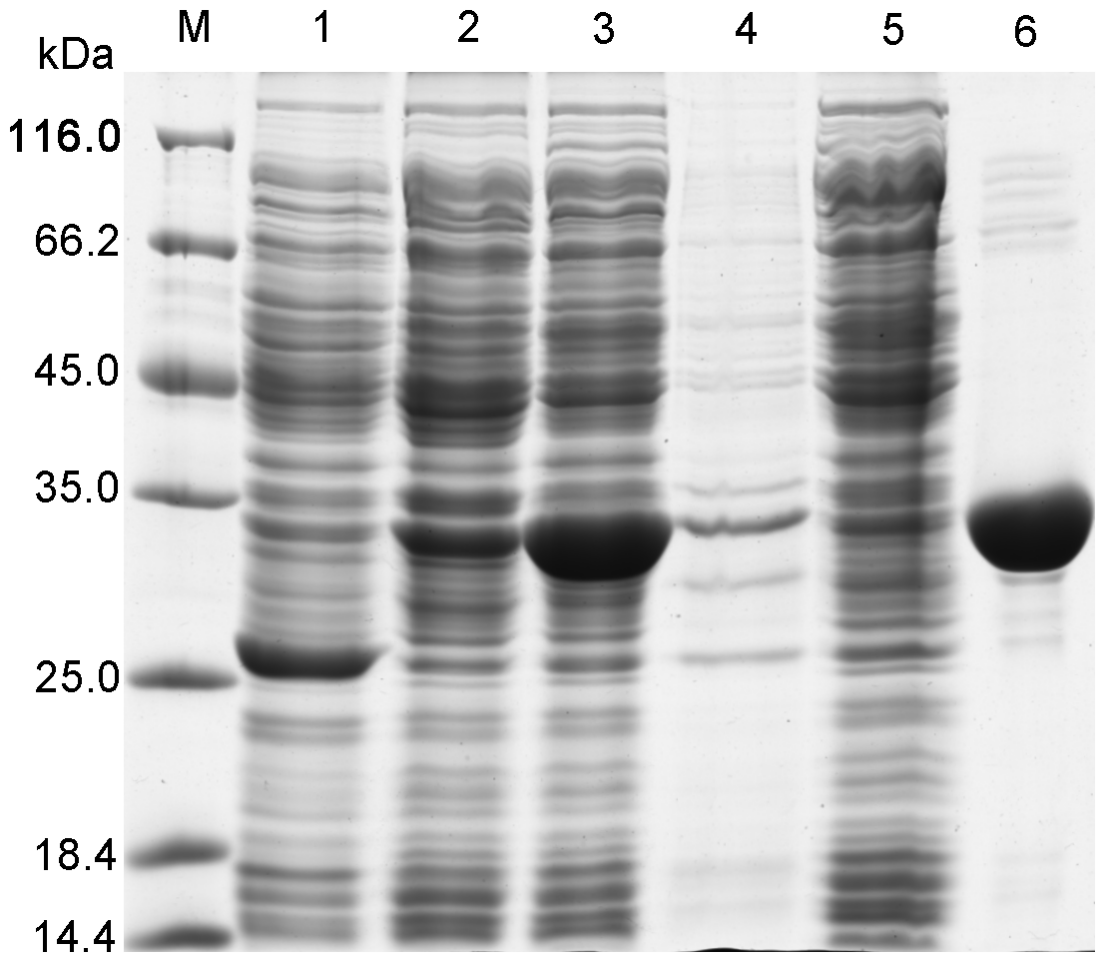
SDS-PAGE analysis of the expression and purification of DadB. M, marker; lane 1, supernatant of *E. coli* BL21(DE3) without any vectors; lane 2, supernatant before induction; lane 3: supernatant after induction; lane 4, resuspended precipitate; lane 5, flow-through after binding; and lane 6, purified DadB.

As shown in Figure 1, most of the DadB expressed in *E. coli* BL21(DE3) was soluble rather than in inclusion bodies and accounted for 48% of the total soluble protein in the supernatant. DadB efficiently bound to the affinity column of Ni Sepharose 6 Fast Flow. After one-step purification by nickel ion affinity, the purity of DadB reached approximately 97%. Based on Figure 1, the molecular weight of denatured DadB monomer was calculated as 32.5 kDa, which is very close to the theoretical molecular weight of 34.2 kDa, calculated according to the amino acid sequence. Finally, the yield of DadB purification was 20 mg per g of wet cell mass. The concentration of purified DadB was measured as 18.6 mg/mL using the Bradford method with bovine serum albumin as the standard sample [40].

### Activity profile of DadB

The dehalogenation activity of DadB was assessed for 46 substrates with a procedure that was described elsewhere [18]. DadB dehalogenated a variety of halogenated compounds, including chlorinated, brominated, and iodinated alkanes; cycloalkanes; alkenes; ethers; and nitriles with chain length ranging from C_1_ to C_14_ (Table 1). Among the tested substrates, DadB showed high activity toward 1,2,3-trichloropropene, 3-chloro-2-methylprop-1-ene, 1,2,3-tribromopropane, 4-bromobutanenitrile, 1,2-dibromoethane, 2,3-dichloroprop-1-ene, and 1,3-dichloropropene.

**Table 1:** Specific activity (nmol·s^−1^·mg^−1^) of DadB and comparison with other HLDs.

Substrates	Specific activity (nmol·s^−1^·mg^−1^)					
	DadB	LinB^a^	DbjA^a^	DhaA^a^	DhlA^a^	DmbA^a^
Group A						
1-Chlorobutane	18.5±0.4	23.1	13.3	12.8	11.7	17.1
1-Bromobutane	75.2±2.5	48.9	29.7	11.6	19.9	6.6
1-Chlorohexane	12.7±1.0	27.0	37.0	6.5	1.3	2.9
1-Bromohexane	14.3±0.2	29.3	24.5	13.9	29.2	1.8
1,5-Dichloropentane	14.4±1.0	28.8	33.4	8.6	2.1	5.7
Group B						
1,2-Dichloroethane	11.7±0.4	ND	8.4	1.1	66.7	ND
1,2-Dibromoethane	236.0±10.8	133.4	92.8	64.8	64.3	41.9
1-Bromo-2-chloroethane	192.2±31.1	94	49.3	74.9	72.5	45.2
1,3-Dichloropropane	88.9±2.9	20.4	32.3	21.8	50.4	28.3
1,2-Dichloropropane	5.2±0.1	ND	3.5	ND	ND	ND
1,3-Dibromopropane	172.4±3.0	92.5	69.7	20	45.1	9.3
1,2-Dibromopropane	124.6±2.1	62.5	19.7	36.5	23.6	0.6
1-Bromo-3-chloropropane	165.3±0.8	86.0	67.0	22.2	38.4	15.8
2-Bromo-1-chloropropane	138.4±9.8	59.9	419.7	19.5	17.6	18.5
1,2,3-Trichloropropane	5.6±0.6	ND	4.5	1.8	ND	ND
1,2,3-Tribromopropane	252.5±26.1	93.6	40.4	49.7	5.9	29.9
1,2-Dibromo-3-chloropropane	135.7±3.6	ND	ND	45.1	5.7	ND
Group C						
1-Iodopropane	64.7±2.1	66.5	75	22.8	14.1	31.8
1,3-Diiodopropane	40.0±3.2	47.9	44.4	39.1	28.6	ND
2-Iodobutane	NA	10.1	33.9	7	4	154.4
1-Iodobutane	50.6±6.8	56.5	56	14.8	13.6	7.9
1-Iodohexane	15.4±1.2	46	45.7	12	13.9	2.9
Chlorocyclopentane	59.0±1.5	5.9	22.3	5.3	2.9	22.7
Chlorocyclohexane	ND	7.4	5.7	0.7	ND	ND
Bromocyclohexane	24.6±2.4	24.9	15	2.3	17.2	3.1
(Bromomethyl)cyclohexane	4.6±0.01	8.5	0	2.3	3.7	0
1-Chloro-2-(2-chloroethoxy)ethane	60.5±1.3	17.7	16.3	9.1	ND	87.5
3-Chloro-2-methylprop-1-ene	400.9±11.9	35.1	57.2	15.5	38	19.9
2,3-Dichloroprop-1-ene	234.2±9.5	15.5	53.6	23.9	62.3	22.5
4-Bromobutanenitrile	240.7±8.3	57.8	77.3	39.6	63.3	7.9
Group D						
Dichloromethane	1.0±0.1					
1-chloro-2-methylpropane	0.4±0.1					
1,3-dichloropropene	225.7±5.2					
1,2,3-trichloropropene	441.3±40.2					
1-Chlorooctane	21.1±2.0					
1-Chlorodecane	1.4±0.4					
1-Chlorododecane	0.3±0.04					
1-Chlorotetradecane	0.2±0.02					
1-Chlorohexadecane	ND					
1-Bromohexadecane	ND					
1-Chlorooctadecane	ND					
Trichloromethane	ND					
1-Chloro-3-nitrobenzene	ND					
4-Bromodiphenyl ether	ND					
Decabromodiphenyl	ND					
Trichloroacetic acid	ND					

Group A, chlorinated or brominated alkanes longer than C_3_; Group B, chlorinated or brominated C_2_ and C_3_ alkanes; Group C, iodinated alkanes and halogenated cycloalkanes, alkenes, ethers, nitriles; Group D, other substrates used in activity assay of DadB. ND, no detectable activity. NA, no available activity. 2-Iodobutane is unstable and its spontaneous dehalogenation interferes the detection of iodine ion concentration seriously, so accurate activity to 2-iodobutane was not obtained.

a The activity data of these five HLDs were collected from the paper of Koudelakova et al. [18].

DadB preferred brominated substrates over than their chlorinated and iodinated counterparts (Table 1 and Figure S3 in File S1). For example, its activities against brominated C_1_-C_4_ substrates are generally 20 times higher (2-40 times) than the corresponding chlorinated substrates. In the case of halogenated butane, the activities were 1-bromobutane>1-iodobutane>1-chlorobutane. For 1,3-dihalogenated propane, the activities were 1,3-dibromopropane>1-chloro-3-bromopropane >1,3-dichloropropane >1,3-diiodopropane. The activity of DadB toward the four iodinated substrates was lower than DbjA and LinB, while the activity of DadB toward brominated substrates was generally higher than the other 5 HLDs (Table 1).

In addition, the activity of DadB against chlorinated alkenes, such as 2,3-dichloroprop-1-ene, 3-chloro-2-methylprop-1-ene, 1,3-dichloropropene, and 1,2,3-trichloropropene, was higher than the corresponding chlorinated alkanes (Table 1 and Figure S3 in File S1). As far as we know, DadB is the first HLD that prefers chlorinated alkenes.

Compared with other HLDs, DadB preferred small (C_2_ and C_3_) substrates. Its activity was 2-4 times that of LinB toward all 12 halogenated propanes and ethanes among the 30 typical substrates, even though it shared the highest sequence similarity (62%) with LinB from among the identified HLDs. Moreover, DadB showed the highest activity toward these substrates even among all 6 HLDs that had enzymatic data available, except for the activities of DhlA against 1,2-dichloroethane and DbjA against 2-bromo-1-chloropropane. Surprisingly, DadB was also active against dichloromethane, which has not been reported for other HLDs. Although DhlA also preferred small substrates [11], [18], its activity toward C_2_-C_3_ substrates was lower than DadB except in the case of 1,2-dichloroethane.

Interestingly, DadB showed activity against 1,2-dichloroethane, 1,2-dichloropropane and 1,2,3-trichloropropane (Table 1), which are persistent environmental pollutants [6]. In contrast, no other HLDs except DbjA can degrade all three of these compounds. The first compound can only be degraded by DhlA, DbjA and DhaA; the second can only be degraded by DbjA; and the third can only be degraded by DbjA and DhaA [18], [41]. DadB exhibited greater activity against these three compounds than other HLDs, including DbjA, except DhlA toward 1,2-dichloroethane.

DadB also can dehalogenate some substrates with longer chain lengths (Table 1, group D). Generally, the activity of DadB decreased when the chain length increased from C_3_, with the exceptions of 1-chlorooctane, which showed higher activity (21.14 nmol·s^−1^·mg^−1^) than 1-chlorohexane (12.66 nmol·s^−1^·mg^−1^) and 1-chlorobutane (18.46 nmol·s^−1^·mg^−1^). The activities toward 1-halogenated butanes and 1-halogenated hexanes were 1-chlorobutane>1-chlorohexane, 1-bromobutane>1-bromohexane, and 1-iodobutane>1-iodohexane. DadB showed low activity against 1-chlorodecane (1.43 nmol·s^−1^·mg^−1^), 1-chlorododecane (0.29 nmol·s^−1^·mg^−1^) and 1-chlorotetradecane (0.21 nmol·s^−1^·mg^−1^), while no activity was detected against 1-chlorohexadecane and 1-chlorooctadecane.

However, DadB showed no advantage against C_4_ and longer substrates compared with the other identified HLDs (Table 1, group A and group D). In the case of 1-chlorobutane, 1-chlorohexane, 1,5-dichloropentane, and 1-bromohexane, DadB showed lower activities than LinB and some additional enzymes. Among the substrates that were longer than C_3_, DadB only showed higher activity against 1-bromobutane than the other 5 HLDs.

DadB possessed the highest overall activity among the previously identified HLDs (Table 1 and Figure S4 in File S1), even higher than LinB and DbjA, which had the highest activities prior to this report [18]. In addition, DadB showed broad substrate specificity. Among the previously characterized HLDs, DhaA has the widest substrate range, and only 1,2-dichloropropane cannot be dehalogenated from among the 30 typical substrates [18], [41]. Similarly, for DadB, only one substrate, chlorocyclohexane, from among the 30 typical substrates cannot be dehalogenated, but it can be degraded by LinB (7.4 nmol·s^−1^·mg^−1^), DbjA (5.7 nmol·s^−1^·mg^−1^), and DhaA (0.7 nmol·s^−1^·mg^−1^) [18], [41].

### Effects of temperature and pH on activity of DadB

The effect of temperature on DadB's activity was investigated with 1,3-dibromopropane (Figure S5 in File S1). Its activity increased gradually from 20°C to 50°C and decreased above 50°C, with the temperature optimum being 50°C. DadB sharply lost activity at 60°C. To obtain its pH optimum, we determined the catalytic activity of DadB in different buffers within the range of pH 4.0 to 10.0 (Figure S5 in File S1). The enzyme was active from pH 5.0 to 10.0, but its activity varied in different buffers even at the same pH value. Phosphate and MOPS buffers repressed its activity. In contrast, imidazole buffers promoted its activity, in agreement with the defined mechanism that HLDs need an imidazole group from histidine to activate a water molecule during hydrolysis step [10]. Moreover, DadB showed two activity peaks, one each in the acidic range (pH 5.5-pH 6.0) and alkaline range (pH 8.0–pH 9.0), which is similar to DmbA [28].

### Steady-state kinetic constants of DadB

The kinetic constants of DadB were determined with 6 substrates (Table 2). In general, the *K*_m_ and *k*_cat_ values of DadB were comparable with those of other HLDs.

**Table 2:** Kinetic parameters of DadB and other HLDs.

Substrates	HLD	*K*_m_(mM)	*k*_cat_(s^−1^)	*k*_cat_/*K*_m_(mM^−1^•s^−1^)	*k*_i_(mM)
1-Chlorobutane	DbeA^a^	3.23	0.17	0.05	
	DhaA^a^	0.4	0.86	2.15	
	DhlA^a^	2.2	1.5	0.68	
	DmbA^a^	1.56	0.6	0.38	
	DmbA^b^	0.16±0.04	0.08±0.004	0.5	
	DbjA^c^	4.0±1.8	1.4±0.42	0.35	
	LinB^d^	0.18±0.02	1.6±0.06	8.8	
	LinB^e^	0.23±0.04	1.11±0.05	4.83	
	**DadB**	**4.88**±**0.35**	**1.17**±**0.10**	**0.24**	
1,3-Dibromopropane	LinB^b^	24.1±3.23	40.9±5.2	1.7	0.49±0.06
	DmbA^b^	4.52±0.71	9.20±1.17	2.03	2.65±0.49
	DbjA^c^	0.22±0.07	3.6±0.49	16	
	**DadB**	**0.82**±**0.04**	**13.39**±**0.99**	**16.43**	
1,2-Dibromoethane	LinB^e^	5.54±0.49	29.33±1.19	5.29	
	**DadB**	**8.70**±**0.44**	**17.95**±**0.50**	**2.06**	
4-bromobutanenitrile	**DadB**	**8.47**±**1.27**	**30.80**±**2.49**	**3.64**	**17.93**±**1.97**
3-chloro-2-methylprop-1-ene	DbjA^c^	0.47±0.29	3.5±1.1	7.4	
	**DadB**	**11.33**±**0.99**	**34.26**±**1.99**	**3.02**	
2,3-dichloroprop-1-ene	**DadB**	**15.06**±**1.37**	**20.06**±**1.09**	**1.33**	

a Chovancova, 2011(Doctoral dissertation).

b Jesenska et al., 2005.

c Sato et al., 2005.

d Nagata et al., 1999.

e Chovancova, 2003.

DadB had a larger turnover number *k*_cat_ for brominated substrates and chlorinated alkenes than for 1-chlorobutane (Table 2), which is in agreement with its preferences to brominated substrates and chlorinated alkenes. Its *k*_cat_ values decreased in the order of 3-chloro-2-methylprop-1-ene>4-bromobutanenitrile>2,3-dichloroprop-1-ene>1,2-dibromoethane>1,2-dibromopropane>1-chlorobutane, which is consistent with the order of decreased activity of DadB against these substrates.

Among the 6 substrates, DadB showed the best affinity and the highest *k*_cat_/*K*_m_ constant toward 1,3-dibromopropane (*K*_m_ = 0.82 mM, *k*_cat_/*K*_m_ = 16.43 mM^−1^·s^−1^) (Table 2), which agreed with its preference for small and brominated substrates. DadB showed weak affinity for chlorinated alkenes, even though it had higher activity against them. Its *k*_cat_/*K*_m_ for 1-chlorobutane was 0.24 mM^−1^·s^−1^, which is less than many other identified HLDs and is consistent with DadB having no advantage in converting substrates of C_4_ and longer compared with other identified HLDs (Table 1).

The *k*_cat_ and *k*_cat_/*K*_m_ of DadB toward 1,3-dibromopropane, 1,2-dibromoethane, 4-bromobutanenitrile, 3-chloro-2-methylprop-1-ene and 2,3-dichloroprop-1-ene are at the same magnitude or even higher than DhlA toward 1,2-dichloroethane (*k*_cat_ = 3.3 s^−1^, *k*_cat_/*K*_m_ = 6.2 mM^−1^·s^−1^) (Table 2). The microbes that encode DhlA are effective at removing 1,2-dichloroethane in a full-scale groundwater purification plant [31].

In addition, apparent substrate self-inhibition occurred with 4-bromobutanenitrile, with an inhibition constant of 17.93 mM in the case of DadB. Self-inhibition has also been observed for some other HLDs [28], [30].

### Effects of pH on the stability of DadB

**Figure 2:**
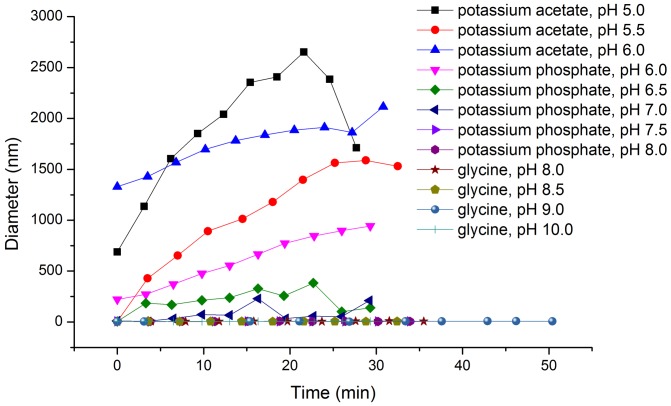
Time-varying particle size of DadB in different buffers. The abscissa and ordinate represent the incubation time of DadB in different buffers at 37°C and the particle diameter, respectively.

Because the activity of DadB decreased rapidly under acidic conditions, the dynamic light scattering (DLS) technique was used to analyze the effect of pH on the stability of DadB. The DLS results showed that DadB aggregated in acidic conditions (Figure 2). After a 30 min incubation in the buffers with pH 7.0 and below at 37°C, DadB formed large polymers or aggregates. Because Figure 2 described the variation of particle size against time, the slopes of the curves represent the aggregation rates. In phosphate buffers of pH 6.5 and pH 7.0, aggregation occurred slower than in more acidic buffers and resulted in fine aggregates. The rapid aggregation of DadB in the acidic buffers may underlie the fast inactivation in such buffers. DadB was stable in buffers with pH≥7.5 (Figure 2). The apparent molecular weight of DadB in such buffers was approximately 19.7 kDa by DLS analysis, indicating that DadB was present as a monomer in alkaline buffers. No aggregation occurred during a 30 min incubation at 37°C in either phosphate or glycine buffers with pH≥7.5. DadB remained as a monomer even when incubated for longer than 50 min at 37°C in 100 mM glycine, pH 9.0. Similarly, other HLDs, such as DmbC and DrbA, also show low activity when forming polymers or aggregates, and maintaining these enzymes as monomers is necessary for high activity [29], [30], [42].

### Homology modeling and structural analysis

Based on the secondary structure prediction (Figure S6 in File S1) and homology modeling (Figure S7 in File S1), DadB is composed of a core domain and a cap domain. It has identical catalytic pentad of HLD-II subfamily, which was also revealed by multiple sequence alignment (Figure S1 in File S1). This pentad includes the nucleophile residue D108, the basic residue H271, the acid catalytic residue E132, and the halide-binding residues N37 and W109.

**Figure 3:**
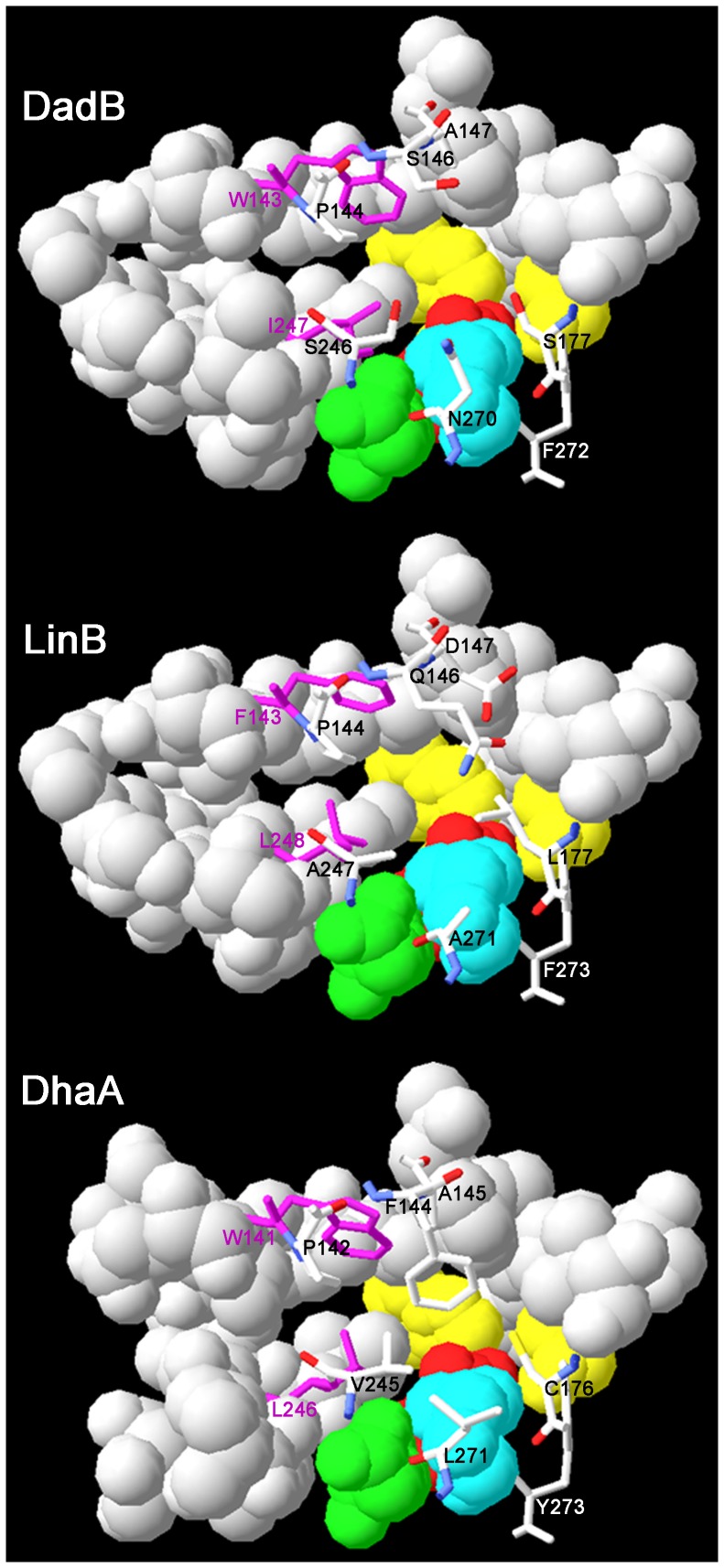
Active sites and access tunnels of LinB, DadB and DhaA. Yellow, cyan, green and red indicate the halide-binding residues, the basic residues, the acidic residues, and the nucleophile residues, respectively. Magenta indicates the two residues at the adjacent position of the main tunnel and the slot tunnel. The pictures were produced with Swiss-PdbViewer 4.04.

The main tunnel of DadB has a larger opening than that of LinB and DhaA (Figure 3). In DadB, the side chains of S146, A147 and S177 are smaller than the corresponding residues in LinB and DhaA. Active site cavities of DadB, LinB and DhaA are 526.8 Å^3^, 322.8 Å^3^ and 261.2 Å^3^, respectively, calculated by CASTp [43].

In general, HLDs with a large active site or a large access tunnel exhibit wider substrate specificity and prefer larger substrates [18], [19], [44]. Water molecules at the active site in HLDs could hinder the substrate binding and lead to low conversion rate toward small substrates [25], [45]. Pavlova et al. found that narrowing the access tunnel of DhaA decreased the accessibility of water molecules to its active site and promoted the formation of activated complex of substrate and enzyme[25]. They obtained a DhaA mutant with increased activity toward 1,2-disubstituted C_2_-C_3_ haloalkanes and decreased activity toward longer (>C_3_) haloalkanes.

Interestingly, DadB possesses a large main tunnel opening, but it prefers small substrates (C_2_ and C_3_) and shows similar activity characteristics as the DhaA mutant [25]. Correspondingly, DadB has two residues (I247 and F272) same as in the DhaA mutant (L246I and Y273F). They may play important role in the preference for small substrates. To evaluate the role of the Phe residue, the following facts should be considered. First, the side chain of the residues in this position (F272 in DadB, F273 in LinB and Y273 in DhaA) are approximately in parallel with the main tunnel instead of pointing into it (Figure 3) [19], [46]. Secondly, the Y273F mutation in DhaA replaced the longer side chain with a shorter one, which could not narrow the tunnel. Therefore, we propose that I247 is crucial in small substrate selection, while F272 in DadB and F273 in DhaA mutant did not contribute to the small substrate selection.

However, it has been unclear how I247 influences the activity characteristics. Oakley et al. proposed that the slot tunnel could provide an exit route for water molecules that exist in the active site cavity [21]. The Ile residue in DadB and DhaA mutant may be more effective at exporting the water molecules from the active site cavity than the corresponding Leu residue in wild-type DhaA and LinB.

Because DadB has relatively high activity against small substrates and narrowing the main tunnel could improve activity against such substrates [25], [47], [48], DadB is an attractive target for protein engineering to degrade small pollutants, such as 1,2,3-trichloropropane.

## Conclusions

Comparisons with other HLDs revealed that DadB can dehalogenate a wide range of substrates but especially prefers short chains, brominated alkanes, and chlorinated alkenes. It has potential for biodegradation and other industrial applications. This is the first report in *Alcanivorax* genus that addresses the function of HLDs. Results of this report strongly support that DadB plays a key role in *A. dieselolei* B-5 to degrade various halogenated alkanes with short chain lengths. Given the key role of *Alcanivorax* bacteria in marine oil bioremediation, strain B-5 can also serve as a cleaner of halogenated alkanes.

## Supporting Information

File S1 **Figure S1**, Multiple sequence alignment of DadA, DadB and structurally described HL**Ds.** The white letters in red background represent identical residues in all HLDs involved in alignment. The red letters in white background indicate similar residues. The secondary structure elements above the sequences come from LinB (1mj5). Residues of catalytic pentad are labeled at the top(▴ indicates the nucleophile residue D, ▪ indicates the catalytic acid residue E of HLD-II members, □ indicates the catalytic acid residue D of HLD-I, • indicates the catalytic base residue H, ♦ indicates the first halide-binding residue W, ★ indicates the second halide-binding residue of HLD-II members, ☆ indicates the second halide-binding residue of HLD-I members. The multiple sequence alignment was conducted by ClustalX2.1[1] and printed by ESPript 2.2[16]. According to this figure, DadA and DadB has typical catalytic pentad of HLD-II members. In DadB, the catalytic pentad includes the nucleophile residue D108, base residue H271, the acid catalytic residue E132, and two halide-binding residues, N37 and W109. So DadB may have dehalogenation activity as identified HLDs with hydrolysis mechanism. **Figure S2, Phylogenetic analyses** of DadA, DadB and other 18 **identified** **HLDs**. Multiple sequence alignment was conducted by MUSCLE[17] and the tree was constructed with Neighbor-Joining method[18] by MEGA 5.05 [19]. Robustness of output trees were estimated by bootstrapping the data 1000 times. This phylogenetic tree is basically the same with the tree of Chovancova (Chovancova et al. 2007). **Figure S3, Substrate specificity profile of DadB toward chlorinated (blue), brominated (red), and iodinated (green) substrates.** The activities of 4 chlorinated alkenes and the corresponding chlorinated alkanes are indicated in the black box. **Figure S4, Activity comparison of DadB with other HLDs.** The values, except for DadB, were obtained from the results published by Koudelakova et al. [20]. Two activities greater than 250 nmol·s^−1^·mg^−1^ are cut off and labeled with the values. **Figure S5, Effect of temperature and pH on the activity of DadB.** Both experiments chose 1,3-dibromopropane as substrate and the data are expressed as relative activities. The data in the left picture are determined in 100 mM glycine buffer, pH 8.6 under different temperatures. The data in the right picture are determined at 37°C in different buffers (▴, 100 mM potassium acetate buffers with pH 4.0, 5.0, 5.5 and 6.0; ▪, 100 mM imidazole buffers with pH 5.5, 6.0, 6.5, 7.0, 7.5, 8.0, 8.5 and 9.0; •, 100 mM MOPS buffers with pH 6.0, 6.5, 7.0, 7.5 and 8.0; ♦, 100 mM potassium phosphate buffers with pH 6.0, 6.5, 7.0, 7.5 and 8.0; ▾, 100 mM glycine buffers with pH 8.0, 8.5, 9.0 and 10.0). **Figure S6, Secondary structure elements prediction of HLDs.** Sequences of DadB and other 7 HLDs with crystal structures [8], [11], [21]–[25] were submitted to PRIPRED server (http://bioinf.cs.ucl.ac.uk/psipred/). And multiple sequence alignment was conducted by ClustalX2.1[1]. The fragments in blue, magenta and yellow background represent the realistic (7 HLDs with solved structures) or predicted (homology modeling of DadB) β-sheets, α- helices and coiled coils respectively. The blue, magenta and yellow letters means they are belong to β-sheets, α- helices and coiled coils according to the Secondary structure elements prediction. **Figure S7, Three-dimensional structure model of DadB.** The cyan and green elements constitute the cap domain and the main domain, respectively. The yellow, red, magenta, blue represents the halide-binding residues, the nucleophile residue, the acid residue, and the base residue, respectively. **Table S1, Halogenated substrates used in the activity determination.** **Table S2, Oligonucleotides used for gene cloning and vector construction.** **Table S3, Conditions used in determination of steady-state kinetic constants** by gas chromatography. **Table S4, Similarity matrix of DadB and other HLDs identified.**
(DOCX)
