# Supplementary material for: Biochemical Characterization of a Haloalkane Dehalogenase DadB from Alcanivorax dieselolei B-5
Source: PLoS One. 2014 Feb 28;9(2):e89144. doi: 10.1371/journal.pone.0089144 (PMC3938430; doi:10.1371/journal.pone.0089144)
Supplement: File S1 — Figure S1, Multiple sequence alignment of DadA, DadB and structurally described HLDs. The white letters in red background represent identical residues in all HLDs involved in alignment. The red letters in white background indicate similar residues. The secondary structure elements above the sequences come from LinB (1mj5). Residues of catalytic pentad are labeled at the top(▴ indicates the nucleophile residue D, ▪ indicates the catalytic acid residue E of HLD-II members, □ indicates the catalytic acid residue D of HLD-I, • indicates the catalytic base residue H, ♦ indicates the first halide-binding residue W, ★ indicates the second halide-binding residue of HLD-II members, ☆ indicates the second halide-binding residue of HLD-I members. The multiple sequence alignment was conducted by ClustalX2.1[1] and printed by ESPript 2.2[16]. According to this figure, DadA and DadB has typical catalytic pentad of HLD-II members. In DadB, the catalytic pentad includes the nucleophile residue D108, base residue H271, the acid catalytic residue E132, and two halide-binding residues, N37 and W109. So DadB may have dehalogenation activity as identified HLDs with hydrolysis mechanism. Figure S2, Phylogenetic analyses of DadA, DadB and other 18 identified HLDs. Multiple sequence alignment was conducted by MUSCLE[17] and the tree was constructed with Neighbor-Joining method[18] by MEGA 5.05 [19]. Robustness of output trees were estimated by bootstrapping the data 1000 times. This phylogenetic tree is basically the same with the tree of Chovancova (Chovancova et al. 2007). Figure S3, Substrate specificity profile of DadB toward chlorinated (blue), brominated (red), and iodinated (green) substrates. The activities of 4 chlorinated alkenes and the corresponding chlorinated alkanes are indicated in the black box. Figure S4, Activity comparison of DadB with other HLDs. The values, except for DadB, were obtained from the results published by Koudelakova et al. [20]. Two activities greater tha [file pone.0089144.s001.docx]

**Supporting Information**

**Table S1** **Halogenated substrates used in the activity determination.**

**Table S2 Oligonucleotides used for gene cloning and vector construction.**

**Table S3** **Conditions used in determination of steady-state kinetic constants by gas chromatography.**

**Table S4** **Similarity matrix of DadB and other HLDs identified.**

**Figure S1** **Multiple sequence alignment** of DadA, DadB and structurally described HL**Ds.** The white letters in red background represent identical residues in all HLDs involved in alignment. The red letters in white background indicate similar residues. The secondary structure elements above the sequences come from LinB (1mj5). Residues of catalytic pentad are labeled at the top(▲ indicates the nucleophile residue D, ■ indicates the catalytic acid residue E of HLD-II members, □ indicates the catalytic acid residue D of HLD-I, ● indicates the catalytic base residue H, ◆ indicates the first halide-binding residue W, ★ indicates the second halide-binding residue of HLD-II members, ☆ indicates the second halide-binding residue of HLD-I members. The multiple sequence alignment was conducted by ClustalX2.1[[1](#_ENREF_1)] and printed by ESPript 2.2[[16](#_ENREF_16)]. According to this figure, DadA and DadB has typical catalytic pentad of HLD-II members. In DadB, the catalytic pentad includes the nucleophile residue D108, base residue H271, the acid catalytic residue E132, and two halide-binding residues, N37 and W109. So DadB may have dehalogenation activity as identified HLDs with hydrolysis mechanism.

**Figure S2** **Phylogenetic analyses** of DadA, DadB and other 18 **identified** **HLDs.** Multiple sequence alignment was conducted by MUSCLE[[17](#_ENREF_17)] and the tree was constructed with Neighbor-Joining method[[18](#_ENREF_18)] by MEGA 5.05 [[19](#_ENREF_19)]. Robustness of output trees were estimated by bootstrapping the data 1000 times. This phylogenetic tree is basically the same with the tree of Chovancova (Chovancova et al. 2007).

**Figure S3 Substrate specificity profile of DadB toward chlorinated (blue), brominated (red), and iodinated (green) substrates.** The activities of 4 chlorinated alkenes and the corresponding chlorinated alkanes are indicated in the black box.

**Figure S4** **Activity comparison of DadB with other HLDs.** The values, except for DadB, were obtained from the results published by Koudelakova et al. [[20](#_ENREF_20)]. Two activities greater than 250 nmol∙s^−1^∙mg^−1^ are cut off and labeled with the values.

**Figure S5** **Effect of temperature and pH on the activity of DadB.** Both experiments chose 1,3-dibromopropane as substrate and the data are expressed as relative activities. The data in the left picture are determined in 100 mM glycine buffer, pH 8.6 under different temperatures. The data in the right picture are determined at 37^o^C in different buffers (▲, 100 mM potassium acetate buffers with pH 4.0, 5.0, 5.5 and 6.0; ■, 100 mM imidazole buffers with pH 5.5, 6.0, 6.5, 7.0, 7.5, 8.0, 8.5 and 9.0; ●, 100 mM MOPS buffers with pH 6.0, 6.5, 7.0, 7.5 and 8.0; ◆, 100 mM potassium phosphate buffers with pH 6.0, 6.5, 7.0, 7.5 and 8.0; ▼, 100 mM glycine buffers with pH 8.0, 8.5, 9.0 and 10.0).

**Figure S6** **Secondary structure elements prediction of HLDs.** Sequences of DadB and other 7 HLDs with crystal structures [[8](#_ENREF_8),[11](#_ENREF_11),[21-25](#_ENREF_21)] were submitted to PRIPRED server (http://bioinf.cs.ucl.ac.uk/psipred/). And multiple sequence alignment was conducted by ClustalX2.1[[1](#_ENREF_1)]. The fragments in blue, magenta and yellow background represent the realistic (7 HLDs with solved structures) or predicted (homology modeling of DadB) β-sheets, α- helices and coiled coils respectively. The blue, magenta and yellow letters means they are belong to β-sheets, α- helices and coiled coils according to the Secondary structure elements prediction.

**Figure S7** **Three-dimensional structure model of DadB.** The cyan and green elements constitute the cap domain and the main domain, respectively. The yellow, red, magenta, blue represents the halide-binding residues, the nucleophile residue, the acid residue, and the base residue, respectively.

**Table S1** **Halogenated substrates used in the activity determination.**

| substrates | brand | purity |
| --- | --- | --- |
| 1-chlorobutane | Sigma-Aldrich | 99.5% |
| 1-chlorohexane | Aldrich | 99% |
| 1-bromobutane | Sigma-Aldrich | 99% |
| 1-bromohexane | Aldrich | 98% |
| 1-iodopropane | Aldrich | 99% |
| 1-iodobutane | Aldrich | 99% |
| 1-iodohexane | Aldrich | ≥98% |
| 1,2-dichloroethane | Fluka | ≥99.5% |
| 1,3-dichloropropane | Aldrich | 99% |
| 1,5-dichloropentane | Aldrich | 99% |
| 1,2-dibromoethane | TCI | >99% |
| 1,3-dibromopropane | Dr.Ehrenstorfer | 100% |
| 1-bromo-3-chloropropane | Sigma | 99% |
| 1,3-diiodopropane | Aldrich | 99% |
| 2-iodobutane | Aldrich | 99% |
| 1,2-dichloropropane | Fluka | ≥99% |
| 1,2-dibromopropane | Aldrich | 97% |
| 2-bromo-1-chloropropane | Dr.Ehrenstorfer | 99% |
| 1,2,3-trichloropropane | Aldrich | 99% |
| 1-chloro-2-(2-chloroethoxy)ethane | Fluka | ≥99%(GC) |
| chlorocyclohexane | Aldrich | 99% |
| bromocyclohexane | Aldrich | 98% |
| (bromomethyl)cyclohexane | Aldrich | 99% |
| 1-bromo-2-chloroethane | Aldrich | 98% |
| chlorocyclopentane | Aldrich | 99% |
| 4-bromobutanenitrile | Alfa Aesar | 97% |
| 1,2,3-tribromopropane | Aldrich | 97% |
| 1,2-dibromo-3-chloropropane | TCI | >98%(GC) |
| 3-chloro-2-methylprop-1-ene | Aldrich | 98% |
| 2,3-dichloroprop-1-ene | Aldrich | 98% |
| dichloromethane | SCRC | ≥99.9% |
| 1-chloro-2-methylpropane | TCI | >95.0%(GC) |
| 1,3-dichloropropene | TCI | >92.0%(GC) |
| 1,2,3-trichloropropene | TCI | >95.0%(GC) |
| 1-chlorooctane | Aldrich | 99% |
| 1-chlorodecane | Aldrich | 98% |
| 1-chlorododecane | Aldrich | ≥97%(GC) |
| 1-chlorotetradecane | Aldrich | 98% |
| 1-chlorohexadecane | Aldrich | 95% |
| 1-bromohexadecane | Aldrich | 97% |
| 1-chlorooctadecane | Aldrich | 96% |
| trichloromethane | SCRC | ≥99.8% |
| 1-chloro-3-nitrobenzene | Aldrich | >98% |
| 4-bromodiphenyl ether | Aldrich | 99% |
| decabromodiphenyl | Aldrich | 98% |
| trichloroacetic acid | Sigma | >99% |

**Table S2 Oligonucleotides used for gene cloning and vector construction.**

| Primer | gene | vector | Sequence (5'-3') | enzyme |
| --- | --- | --- | --- | --- |
| 22b4127-f | *dadB* | pET22b | AAGGAGATATACATATGCTCAGAGAACAACTCCCC | NdeI |
| 22b4127-r | *dadB* | pET22b | GGTGGTGGTGCTCGAGCGAATTGGATAGGGCCT | XhoI |
| 22b2238-f | *dadA* | pET22b | AAGGAGATATACATATGGGCTTCGCGGACTGTCC | NdeI |
| 22b2238-r | *dadA* | pET22b | GGTGGTGGTGCTCGAGTCGCGGATTCGCCAAGCG | XhoI |
| 32a2238-f | *dadA* | pET32a | GGTGCCACGCGGATCCATGGGCTTCGCGGACTGTC | BamHI |
| 32a2238-r | *dadA* | pET32a | GCTCGAATTCGGATCCTCATCGCGGATTCGCCAAGCG | BamHI |
| 28a2238-f | *dadA* | pET28a | CGCGCGGCAGCCATATGGGCTTCGCGGACTGTCC | NdeI |
| 28a2238-r | *dadA* | pET28a | GTCATGCTAGCCATATGTTATCGCGGATTCGCCAAG | NdeI |
| pGEX2238-f | *dadA* | pGEX-4T-1 | AAGGAGATATACATATGCTCAGAGAACAACTCCCC | BamHI |
| pGEX2238-r | *dadA* | pGEX-4T-1 | GGTGGTGGTGCTCGAGCGAATTGGATAGGGCCT | BamHI |

**Table S3** **Conditions used in determination of** **steady-state kinetic constants by gas chromatography.**

| substrates | column temperature (^o^C) | testing time (min) |
| --- | --- | --- |
| 1-chlorobutane | 40 | 4 |
| 1,3-dibromopropane | 130 | 5 |
| 1,2-dibromoethane | 100 | 5 |
| 4-bromobutanenitrile | 170 | 5 |
| 3-chloro-2-methylprop-1-ene | 35 | 5 |
| 2,3-dichloroprop-1-ene | 60 | 6 |

**Table S4** **Similarity matrix of DadB and other HLDs identified.**

| DmbA | 100 |  |  |  |  |  |  |  |  |  |  |  |  |  |  |  |  |  |  |  |
| --- | --- | --- | --- | --- | --- | --- | --- | --- | --- | --- | --- | --- | --- | --- | --- | --- | --- | --- | --- | --- |
| DmsA | 70 | 100 |  |  |  |  |  |  |  |  |  |  |  |  |  |  |  |  |  |  |
| LinB | 69 | 63 | 100 |  |  |  |  |  |  |  |  |  |  |  |  |  |  |  |  |  |
| DadB | 56 | 53 | 60 | 100 |  |  |  |  |  |  |  |  |  |  |  |  |  |  |  |  |
| DbjA | 40 | 39 | 41 | 40 | 100 |  |  |  |  |  |  |  |  |  |  |  |  |  |  |  |
| DbeA | 42 | 41 | 45 | 43 | 73 | 100 |  |  |  |  |  |  |  |  |  |  |  |  |  |  |
| DmlA | 40 | 41 | 46 | 41 | 60 | 61 | 100 |  |  |  |  |  |  |  |  |  |  |  |  |  |
| DhaA | 45 | 44 | 49 | 48 | 51 | 50 | 52 | 100 |  |  |  |  |  |  |  |  |  |  |  |  |
| DmmA | 42 | 43 | 46 | 44 | 43 | 46 | 44 | 50 | 100 |  |  |  |  |  |  |  |  |  |  |  |
| Jann2620 | 43 | 43 | 46 | 42 | 44 | 47 | 44 | 48 | 50 | 100 |  |  |  |  |  |  |  |  |  |  |
| DatA | 35 | 35 | 35 | 32 | 35 | 36 | 36 | 34 | 37 | 35 | 100 |  |  |  |  |  |  |  |  |  |
| Sav4779 | 42 | 44 | 47 | 41 | 41 | 45 | 46 | 46 | 42 | 39 | 44 | 100 |  |  |  |  |  |  |  |  |
| DadA | 35 | 34 | 32 | 34 | 35 | 36 | 34 | 37 | 36 | 37 | 34 | 32 | 100 |  |  |  |  |  |  |  |
| DhlA | 21 | 22 | 25 | 22 | 21 | 21 | 21 | 26 | 22 | 26 | 21 | 22 | 24 | 100 |  |  |  |  |  |  |
| DppA | 23 | 25 | 25 | 24 | 24 | 23 | 26 | 28 | 25 | 26 | 22 | 24 | 25 | 52 | 100 |  |  |  |  |  |
| DhmA | 23 | 24 | 25 | 21 | 22 | 22 | 24 | 27 | 26 | 22 | 22 | 22 | 27 | 36 | 38 | 100 |  |  |  |  |
| DmbB | 22 | 24 | 25 | 22 | 23 | 22 | 24 | 27 | 26 | 23 | 23 | 24 | 26 | 36 | 39 | 83 | 100 |  |  |  |
| DpcA | 21 | 24 | 21 | 20 | 22 | 22 | 22 | 25 | 27 | 26 | 23 | 21 | 26 | 38 | 43 | 50 | 50 | 100 |  |  |
| DmbC | 27 | 27 | 29 | 27 | 27 | 25 | 27 | 27 | 31 | 27 | 22 | 26 | 30 | 20 | 23 | 26 | 24 | 24 | 100 |  |
| DrbA | 27 | 25 | 28 | 25 | 22 | 24 | 26 | 25 | 23 | 24 | 24 | 24 | 25 | 21 | 22 | 21 | 22 | 22 | 28 | 100 |

Values in the table represent the similarity (percentage) of amino acid sequences between the two HLDs at top and left. The bold numbers is the values of DadB. This matrix is produced by ClustalX 2.1[[1](#_ENREF_1),[2](#_ENREF_2)]. LinB(P51698), *Spinghobium japonicum* UT26[[3](#_ENREF_3)]; DmbA(AJ784272), DmbB(AJ784273) and DmbC(AM696288), *Mycobacterium bovis* 5033/66[[4](#_ENREF_4),[5](#_ENREF_5)]; DbjA(NP_767727), *Bradyrhizobium japonicum* USDA110[[6](#_ENREF_6)]; DbeA(BAJ23986), *Bradyrhizobium elkani* USDA94(Prudnikova et al., unpublished data); DmlA(NP_106032), *Mesorhizobium loti* MAFF303099[[6](#_ENREF_6)]; DhaA(AAC15838), *Rhodococcus rhodochrous* NCIMB 13064[[7](#_ENREF_7)]; DmmA(AAT70109), the metagenomic DNA of a marine microbial consortium[[8](#_ENREF_8)]; DatA(AB478945), *Agrobacterium tumefaciens* C58[[9](#_ENREF_9)]; DhlA(AAA88691), *Xanthobacter autotrophicus* GJ10[[10](#_ENREF_10)]; DppA(ZP_01908831), *Plesiocystis pacifica* SIR-1[[11](#_ENREF_11)]; DhmA(AJ314789), *Mycobacterium avium* N85[[12](#_ENREF_12)]; DpcA(YP_580518), *Psychrobacter cryohalolentis* K5[[13](#_ENREF_13)]; DrbA(AM696289), *Rhodopirellula baltica* SH1[[5](#_ENREF_5)]; DmsA(AAL17946), *Mycobacterium smegmatis* ATCC700084[[14](#_ENREF_14)]; Jann2620(YP_510562), *Jannaschia sp.* CCS1[[15](#_ENREF_15)]; Sav4779(NP_825956), *Streptomyces avermitilis MA-4680*[[15](#_ENREF_15)].

**
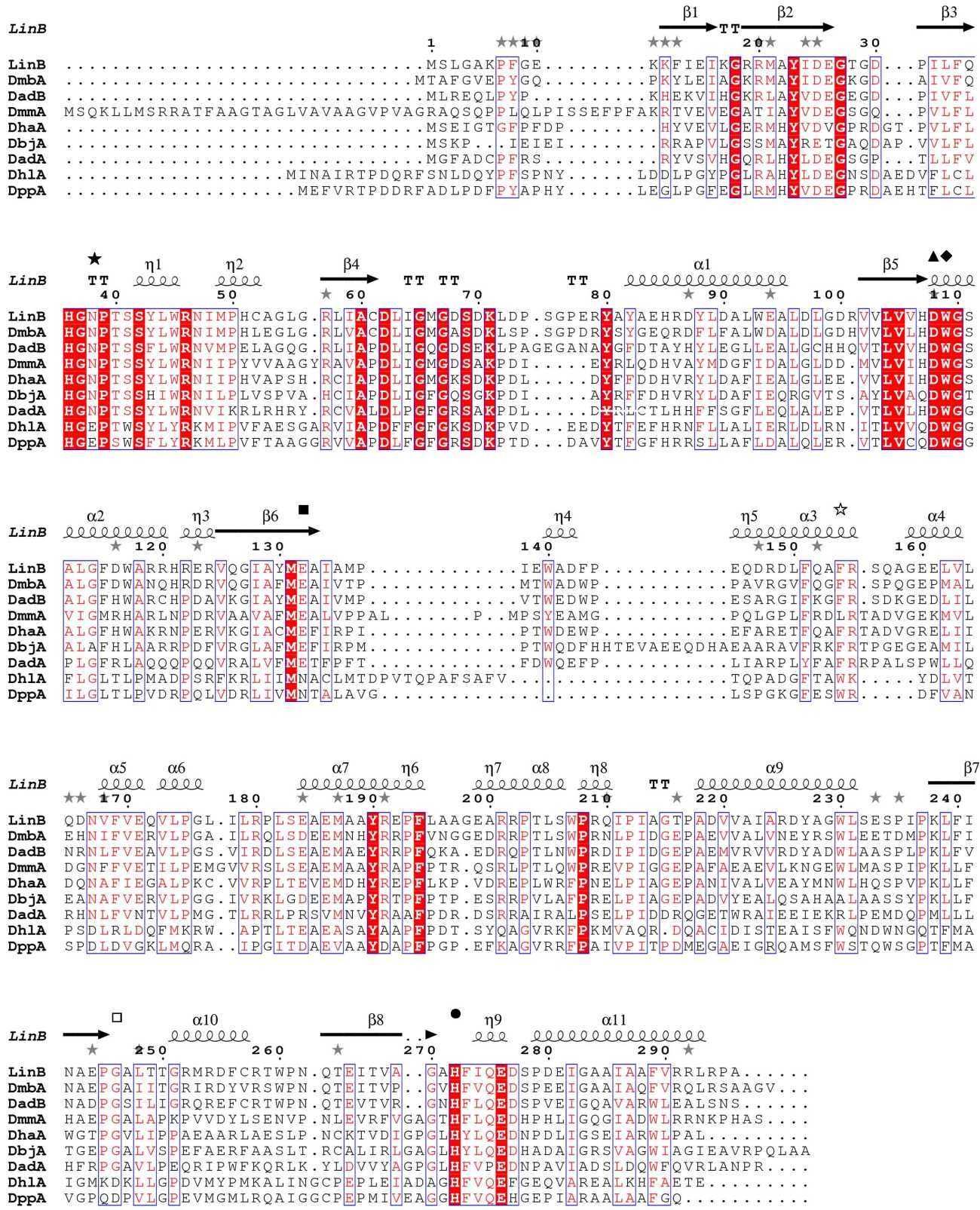
**

**Figure S1** **Multiple sequence alignment** of DadA, DadB and structurally described HL**Ds.** The white letters in red background represent identical residues in all HLDs involved in alignment. The red letters in white background indicate similar residues. The secondary structure elements above the sequences come from LinB (1mj5). Residues of catalytic pentad are labeled at the top(▲ indicates the nucleophile residue D, ■ indicates the catalytic acid residue E of HLD-II members, □ indicates the catalytic acid residue D of HLD-I, ● indicates the catalytic base residue H, ◆ indicates the first halide-binding residue W, ★ indicates the second halide-binding residue of HLD-II members, ☆ indicates the second halide-binding residue of HLD-I members. The multiple sequence alignment was conducted by ClustalX2.1[[1](#_ENREF_1)] and printed by ESPript 2.2[[16](#_ENREF_16)]. According to this figure, DadA and DadB has typical catalytic pentad of HLD-II members. In DadB, the catalytic pentad includes the nucleophile residue D108, base residue H271, the acid catalytic residue E132, and two halide-binding residues, N37 and W109. So DadB may have dehalogenation activity as identified HLDs with hydrolysis mechanism.

**
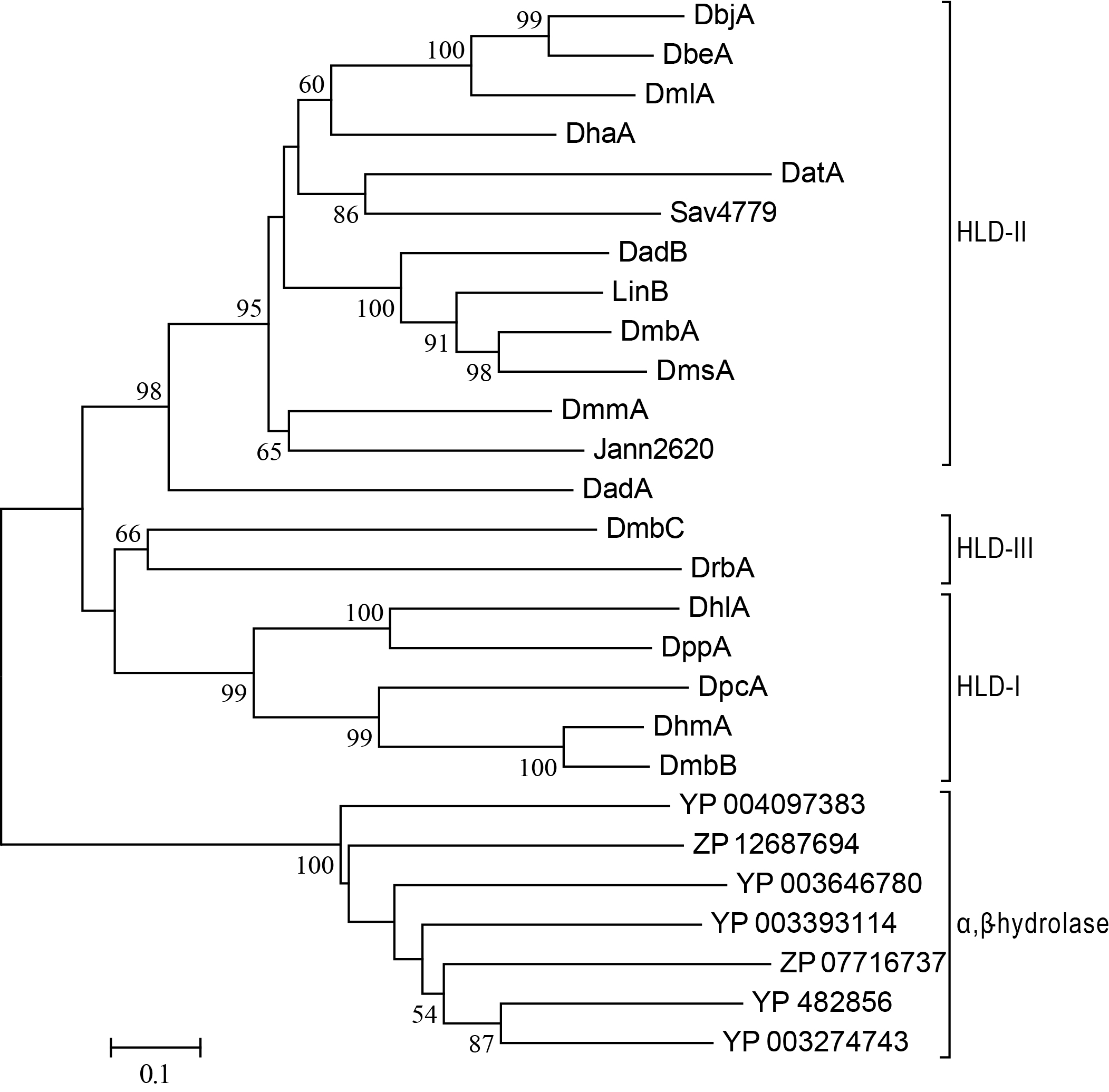
**

**Figure S2** **Phylogenetic analyses** of DadA, DadB and other 18 **identified** **HLDs.** Multiple sequence alignment was conducted by MUSCLE[[17](#_ENREF_17)] and the tree was constructed with Neighbor-Joining method[[18](#_ENREF_18)] by MEGA 5.05 [[19](#_ENREF_19)]. Robustness of output trees were estimated by bootstrapping the data 1000 times. This phylogenetic tree is basically the same with the tree of Chovancova (Chovancova et al. 2007).


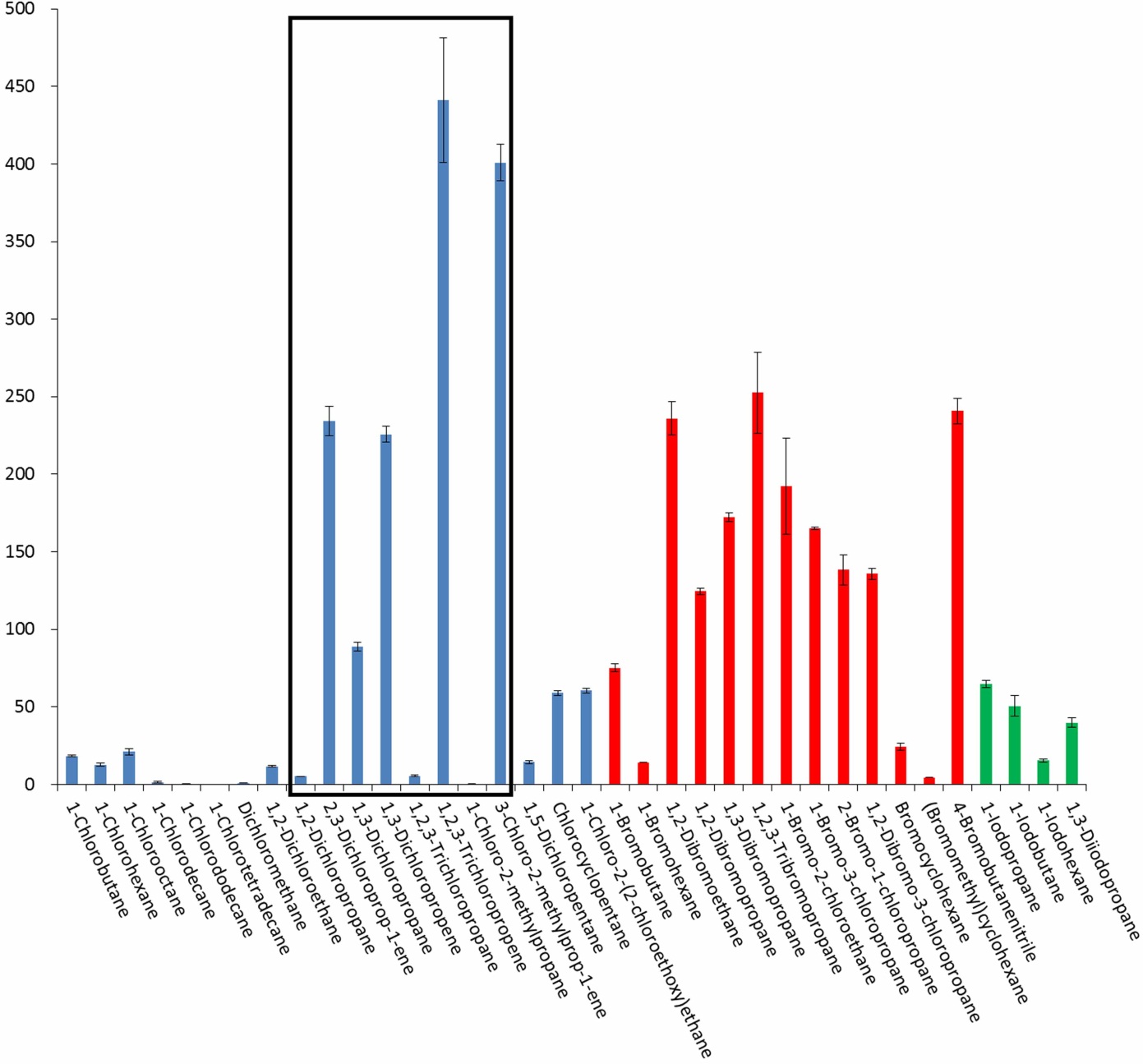


**Figure S3 Substrate specificity profile of DadB toward chlorinated (blue), brominated (red), and iodinated (green) substrates.** The activities of 4 chlorinated alkenes and the corresponding chlorinated alkanes are indicated in the black box.


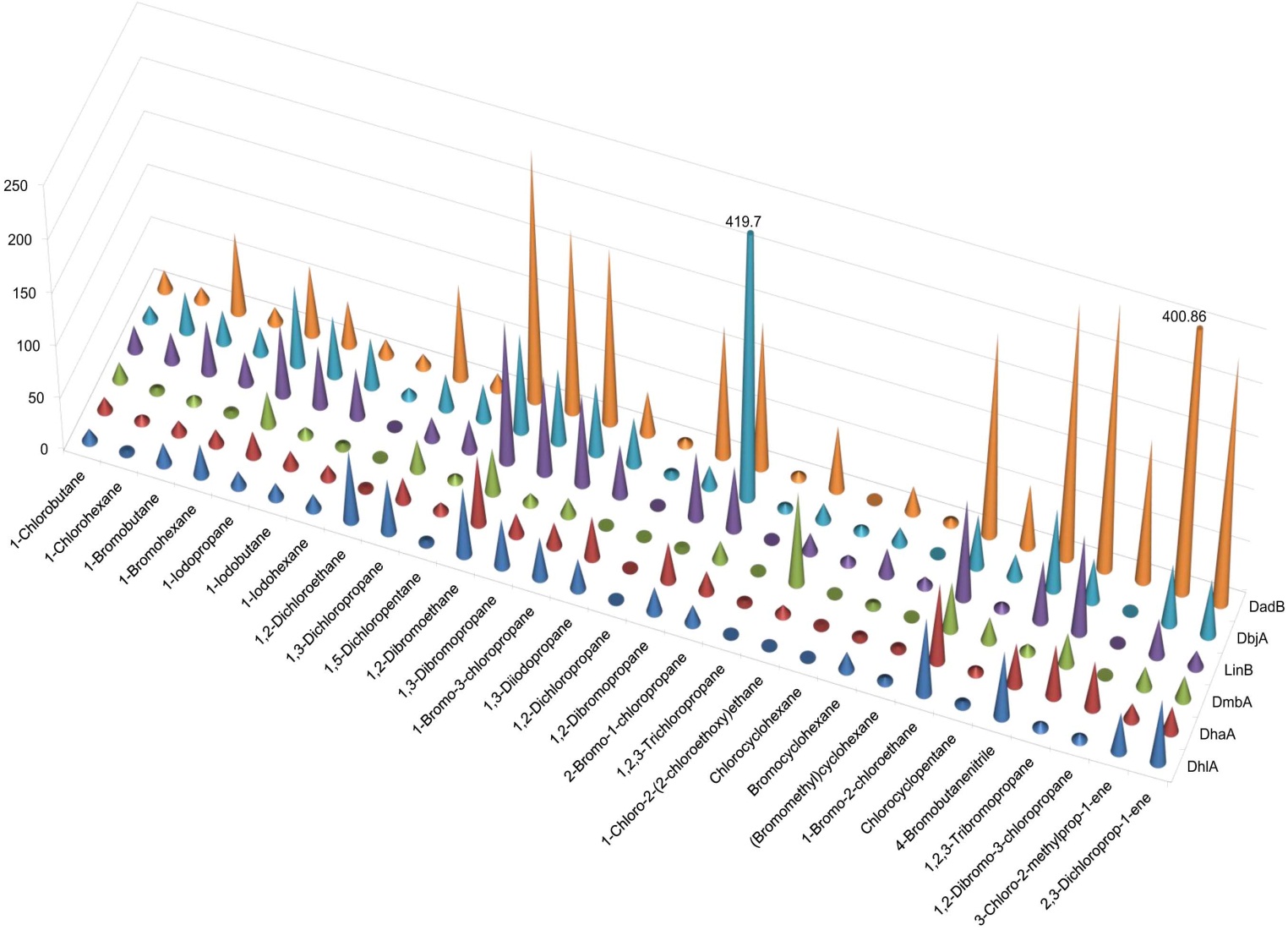


**Figure S4** **Activity comparison of DadB with other HLDs.** The values, except for DadB, were obtained from the results published by Koudelakova et al. [[20](#_ENREF_20)]. Two activities greater than 250 nmol∙s^−1^∙mg^−1^ are cut off and labeled with the values.


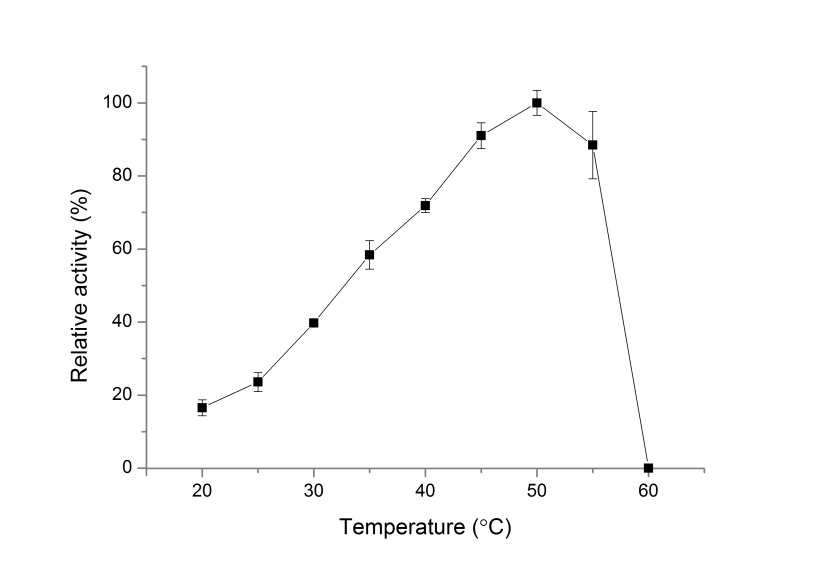

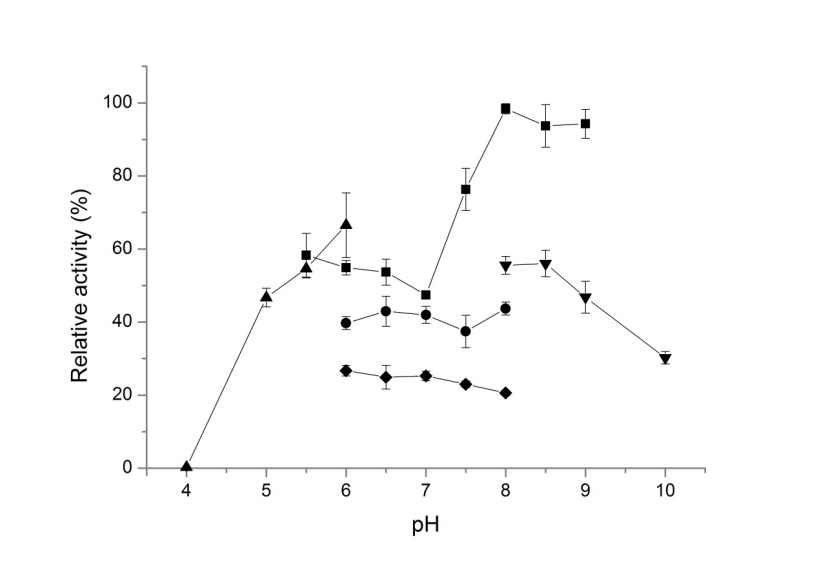


**Figure S5** **Effect of temperature and pH on the activity of DadB.** Both experiments chose 1,3-dibromopropane as substrate and the data are expressed as relative activities. The data in the left picture are determined in 100 mM glycine buffer, pH 8.6 under different temperatures. The data in the right picture are determined at 37^o^C in different buffers (▲, 100 mM potassium acetate buffers with pH 4.0, 5.0, 5.5 and 6.0; ■, 100 mM imidazole buffers with pH 5.5, 6.0, 6.5, 7.0, 7.5, 8.0, 8.5 and 9.0; ●, 100 mM MOPS buffers with pH 6.0, 6.5, 7.0, 7.5 and 8.0; ◆, 100 mM potassium phosphate buffers with pH 6.0, 6.5, 7.0, 7.5 and 8.0; ▼, 100 mM glycine buffers with pH 8.0, 8.5, 9.0 and 10.0).


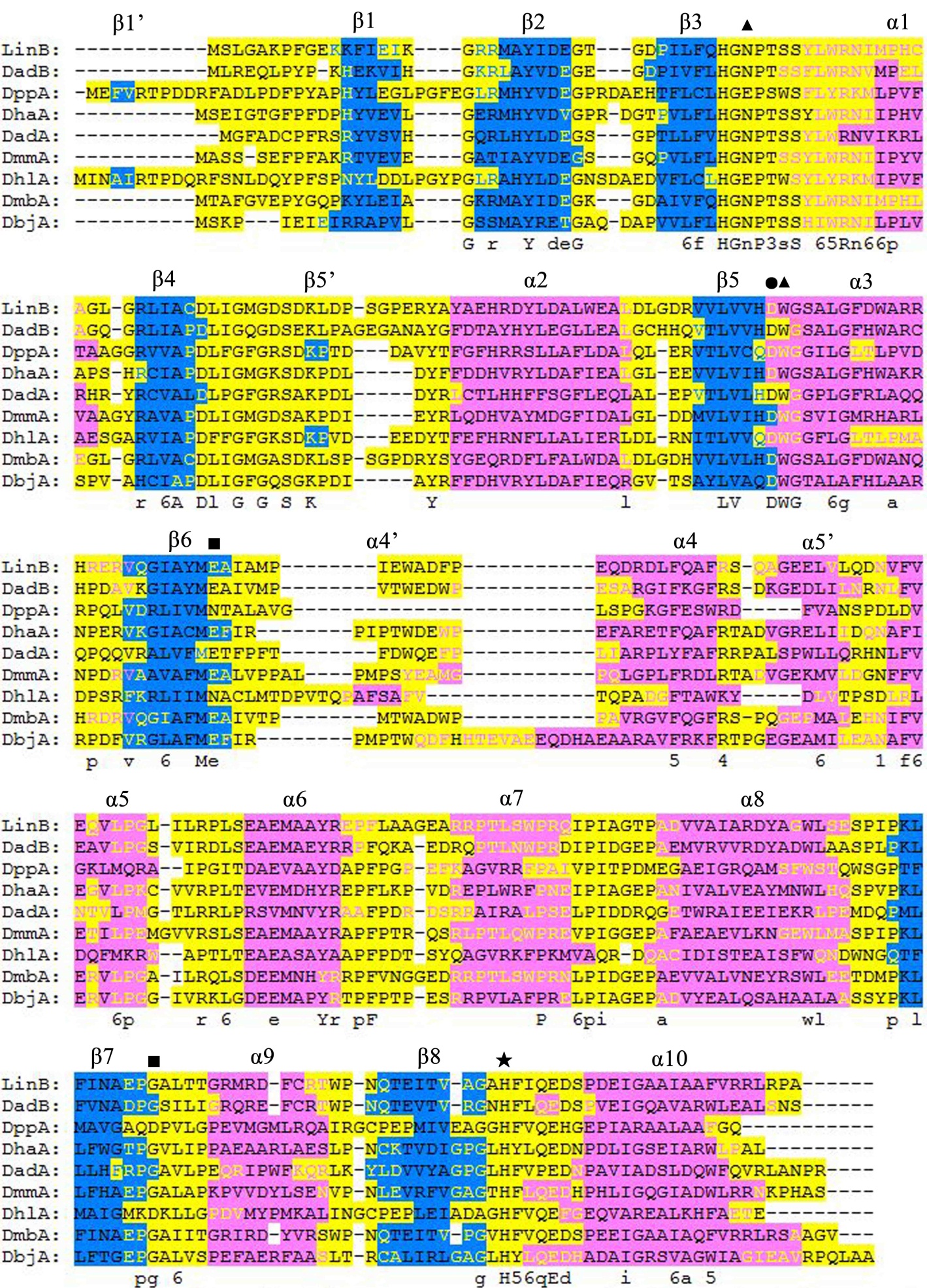


**Figure S6** **Secondary structure elements prediction of HLDs.** Sequences of DadB and other 7 HLDs with crystal structures [[8](#_ENREF_8),[11](#_ENREF_11),[21-25](#_ENREF_21)] were submitted to PRIPRED server (http://bioinf.cs.ucl.ac.uk/psipred/). And multiple sequence alignment was conducted by ClustalX2.1[[1](#_ENREF_1)]. The fragments in blue, magenta and yellow background represent the realistic (7 HLDs with solved structures) or predicted (homology modeling of DadB) β-sheets, α- helices and coiled coils respectively. The blue, magenta and yellow letters means they are belong to β-sheets, α- helices and coiled coils according to the Secondary structure elements prediction.


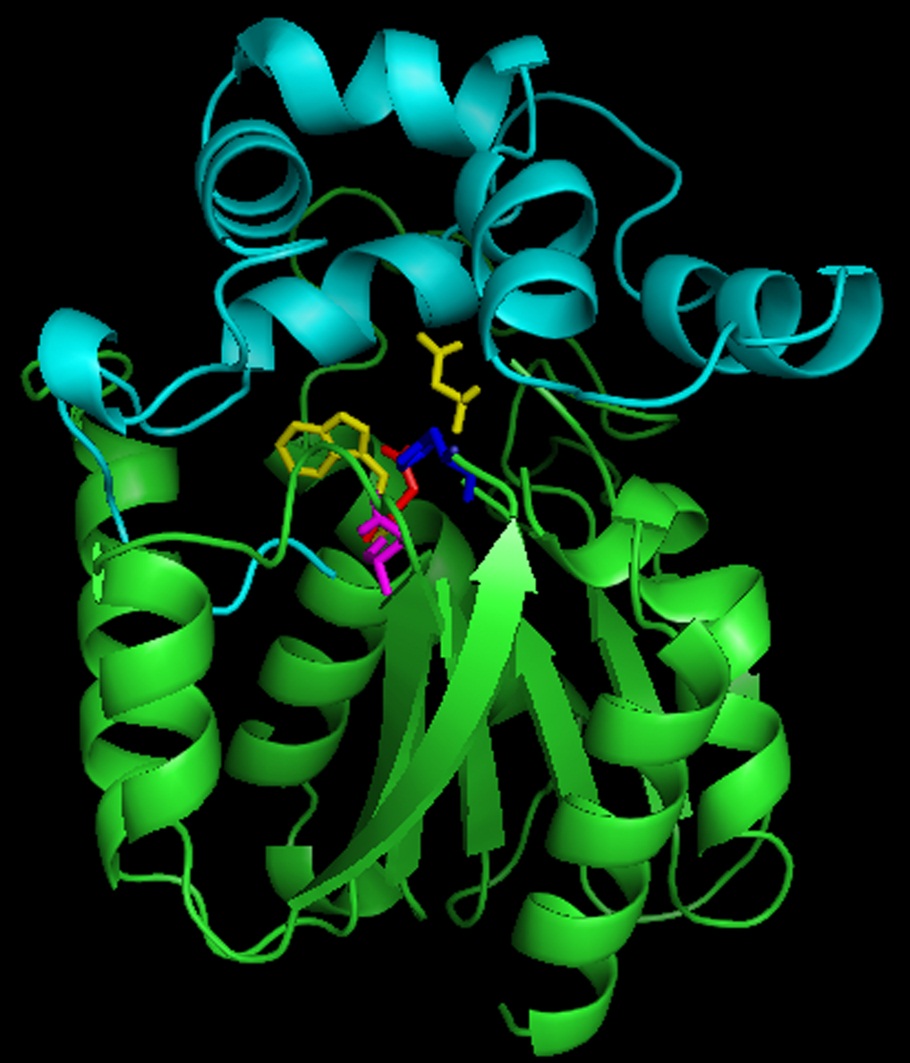


**Figure S7** **Three-dimensional structure model of DadB.** The cyan and green elements constitute the cap domain and the main domain, respectively. The yellow, red, magenta, blue represents the halide-binding residues, the nucleophile residue, the acid residue, and the base residue, respectively.

1. Larkin M, Blackshields G, Brown N, Chenna R, McGettigan P, et al. (2007) Clustal W and Clustal X version 2.0. Bioinformatics 23: 2947-2948.

2. Thompson JD, Gibson T, Higgins DG (2002) Multiple sequence alignment using ClustalW and ClustalX. Current protocols in bioinformatics: 2.3. 1-2.3. 22.

3. Nagata Y, Miyauchi K, Damborsky J, Manova K, Ansorgova A, et al. (1997) Purification and characterization of a haloalkane dehalogenase of a new substrate class from a gamma-hexachlorocyclohexane-degrading bacterium, Sphingomonas paucimobilis UT26. Appl Environ Microbiol 63: 3707-3710.

4. Jesenska A, Pavlova M, Strouhal M, Chaloupkova R, Tesinska I, et al. (2005) Cloning, biochemical properties, and distribution of mycobacterial haloalkane dehalogenases. Appl Environ Microbiol 71: 6736-6745.

5. Jesenska A, Monincova M, Koudelakova T, Hasan K, Chaloupkova R, et al. (2009) Biochemical characterization of haloalkane dehalogenases DrbA and DmbC, Representatives of a Novel Subfamily. Appl Environ Microbiol 75: 5157-5160.

6. Sato Y, Monincova M, Chaloupkova R, Prokop Z, Ohtsubo Y, et al. (2005) Two rhizobial strains, Mesorhizobium loti MAFF303099 and Bradyrhizobium japonicum USDA110, encode haloalkane dehalogenases with novel structures and substrate specificities. Appl Environ Microbiol 71: 4372-4379.

7. Curragh H, Orla Flynn, Michael J L, Thomas M. Stafford, John T. G. Hamilton, et al. (1994) Haloalkane degradation and assimilation by Rhodococcus rhodochrous NCIMB 13064 Microbiology 140: 1433-1442.

8. Gehret JJ, Gu L, Geders TW, Brown WC, Gerwick L, et al. (2011) Structure and activity of DmmA, a marine haloalkane dehalogenase. Protein Science 21: 239-248.

9. Hasan K, Fortova A, Koudelakova T, Chaloupkova R, Ishitsuka M, et al. (2011) Biochemical characteristics of the novel haloalkane dehalogenase DatA, isolated from the plant pathogen Agrobacterium tumefaciens C58. Appl Environ Microbiol 77: 1881-1884.

10. Keuning S, Janssen DB, Witholt B (1985) Purification and characterization of hydrolytic haloalkane dehalogenase from Xanthobacter autotrophicus GJ10. J Bacteriol 163: 635-639.

11. Hesseler M, Bogdanovic X, Hidalgo A, Berenguer J, Palm GJ, et al. (2011) Cloning, functional expression, biochemical characterization, and structural analysis of a haloalkane dehalogenase from Plesiocystis pacifica SIR-1. Appl Microbiol Biotechnol 91: 1049-1060.

12. Jesenska A, Bartos M, Czernekova V, Rychlik I, Pavlik I, et al. (2002) Cloning and expression of the haloalkane dehalogenase gene dhmA from Mycobacterium avium N85 and preliminary characterization of DhmA. Appl Environ Microbiol 68: 3724-3730.

13. Drienovska I, Chovancova E, Koudelakova T, Damborsky J, Chaloupkova R (2012) Biochemical characterization of a novel haloalkane dehalogenase from a cold-adapted bacterium. Appl Environ Microbiol 78: 4995-4998.

14. Chovancova E (2011) Bioinformatic analysis and design of haloalkane dehalogenases [PhD Thesis]: Masaryk University.

15. Chan WY, Wong M, Guthrie J, Savchenko AV, Yakunin AF, et al. (2010) Sequence- and activity-based screening of microbial genomes for novel dehalogenases. Microb Biotechnol 3: 107-120.

16. Gouet P, Courcelle E, Stuart DI (1999) ESPript: analysis of multiple sequence alignments in PostScript. Bioinformatics 15: 305-308.

17. Edgar RC (2004) MUSCLE: multiple sequence alignment with high accuracy and high throughput. Nucleic Acids Res 32: 1792-1797.

18. Saitou N, Nei M (1987) The neighbor-joining method: a new method for reconstructing phylogenetic trees. Mol Biol Evol 4: 406-425.

19. Kumar S, Nei M, Dudley J, Tamura K (2008) MEGA: a biologist-centric software for evolutionary analysis of DNA and protein sequences. Brief Bioinform 9: 299-306.

20. Koudelakova T, Chovancova E, Brezovsky J, Monincova M, Fortova A, et al. (2011) Substrate specificity of haloalkane dehalogenases. Biochemical Journal 435: 345-354.

21. Franken SM, Rozeboom HJ, Kalk KH, Dijkstra BW (1991) Crystal structure of haloalkane dehalogenase: an enzyme to detoxify halogenated alkanes. EMBO J 10: 1297-1302.

22. Newman J, Peat TS, Richard R, Kan L, Swanson PE, et al. (1999) Haloalkane dehalogenases: structure of a Rhodococcus enzyme. Biochemistry 38: 16105-16114.

23. Marek J, Vevodova J, Smatanova IK, Nagata Y, Svensson LA, et al. (2000) Crystal structure of the haloalkane dehalogenase from Sphingomonas paucimobilis UT26. Biochemistry 39: 14082-14086.

24. Mazumdar PA, Hulecki JC, Cherney MM, Garen CR, James MN (2007) X-ray crystal structure of Mycobacterium tuberculosis haloalkane dehalogenase Rv2579. Biochim Biophys Acta 1784: 351-362.

25. Prokop Z, Sato Y, Brezovsky J, Mozga T, Chaloupkova R, et al. (2010) Enantioselectivity of haloalkane dehalogenases and its modulation by surface loop engineering. Angewandte Chemie International Edition 49: 6111-6115.
